# Beyond data and technology: the need for new thinking to enable the era of precision prevention

**DOI:** 10.1186/s12916-026-04938-1

**Published:** 2026-05-14

**Authors:** Maria Tsakiroglou, Pradeep Harish, Harry Hemingway, Sarah P. Blagden, Steve Gardner, Dan O’Connor, Christopher T. Rentsch, Grace Ford, Aris Saoulidis, Rory H. Maclean, Alastair K. Denniston, Munir Pirmohamed

**Affiliations:** 1 Department of Pharmacology and Therapeutics, Institute of Systems Molecular and Integrative Biology, University of Liverpool, Liverpool, UK; 2 Institute of Health Informatics, University College London, London, UK; 3 Oncology Clinical Trials Office (OCTO), Department of Oncology, University of Oxford, Oxford, UK; 4 PrecisionLife Ltd, Oxford, UK; 5 ABPI, London, UK; 6 Faculty of Epidemiology and Population Health, London School of Hygiene & Tropical Medicine, London, UK; 7 Innovate UK, London, UK; 8 Inpatient Pharmacy, Addenbrookes Hospital, Cambridge University Hospitals NHS Foundation Trust, Cambridge, UK; 9 Centre for Rheumatology, Royal Free Campus, Division of Medicine, University College London, London, UK; 10 Academic Unit of Ophthalmology Institute of Inflammation and Ageing, University of Birmingham, Birmingham, UK

**Keywords:** Precision, Prevention, Genomics, AI, Risk stratification, Future healthcare

## Abstract

**Background:**

Global flagship initiatives increasingly advocate for proactive health maintenance to alleviate the growing burden on reactive, disease-focused healthcare systems. Precision prevention is conceived as the targeted modulation of causal pathways across the disease continuum, from latent risk and pre-disease states to clinical manifestation, surpassing conventional public health prevention strategies that prioritise managing population-level risk factors. Traditional discovery and implementation models, however, remain poorly aligned with the pace and breadth of scientific and technological advances. This review outlines key barriers to scaling precision prevention and argues for the integration of conceptual, methodological, and policy perspectives into a single implementation‑oriented framework.

**Main:**

Individualised risk stratification lies at the core of precision prevention. Genomics serves as a stable substrate for lifetime susceptibility assessment, while meaningful prediction in multifactorial chronic disease requires additional risk monitoring using dynamic intermediate molecular markers and high-resolution exposomic data. Machine learning and other artificial intelligence (AI) methods are increasingly helpful tools for integrating large, heterogeneous and temporally structured real-world data to generate personalised predictions of health trajectories. Trustworthy AI-enabled risk prediction or decision-support systems are expected to provide transparency about model logic, assumptions and performance. In discovery, existing diagnostic classifications and conventional case–control designs can obscure mechanistic heterogeneity. Shifting toward precision phenotyping and biologically grounded disease redefinition could reveal a new layer of molecular understanding. Evidence generation strategies that reflect the temporal change of disease, including high‑risk enrichment, surrogate endpoints, and adaptive, trajectory-based monitoring, are particularly important for common conditions with prolonged latency periods (e.g., cancer, cardiovascular disease). Features often dismissed as “noise”, such as stochastic molecular variation and minimal exposures, may in fact encode meaningful individual-level signals and thus merit investigation.

**Conclusion:**

To shift healthcare from reactive treatment toward proactive health maintenance requires coordinated action from stakeholders to reshape the pillars of discovery, reform outcome assessments and modernise implementation strategies.

## Background

A personalised approach to disease management has been central to medical practice since its earliest origins. The Hippocratic Corpus advocated for a holistic approach that placed the individual, and their specific health trajectory and lifestyle (rather than the disease) at the core of healthcare. This contrasts with current health management practice, where diagnosis of common diseases is based on non-specific clinical criteria and therapeutic decisions are inferred from average responses observed in trial cohorts. As a result, our understanding of the efficacy and safety of a treatment derives from a hypothetical individual representing the cohort’s average molecular and clinical response. The advent of precision medicine heralded by advances in biomedical science and computational technologies has equipped us with the tools and infrastructure necessary to translate the patient’s individual clinical context and heterogeneity into routine clinical reality. The growing number of therapies designed to target changes caused by tumour, pathogen or germline variation initially placed genomics at the forefront of modern healthcare.

**Fig. 1:**
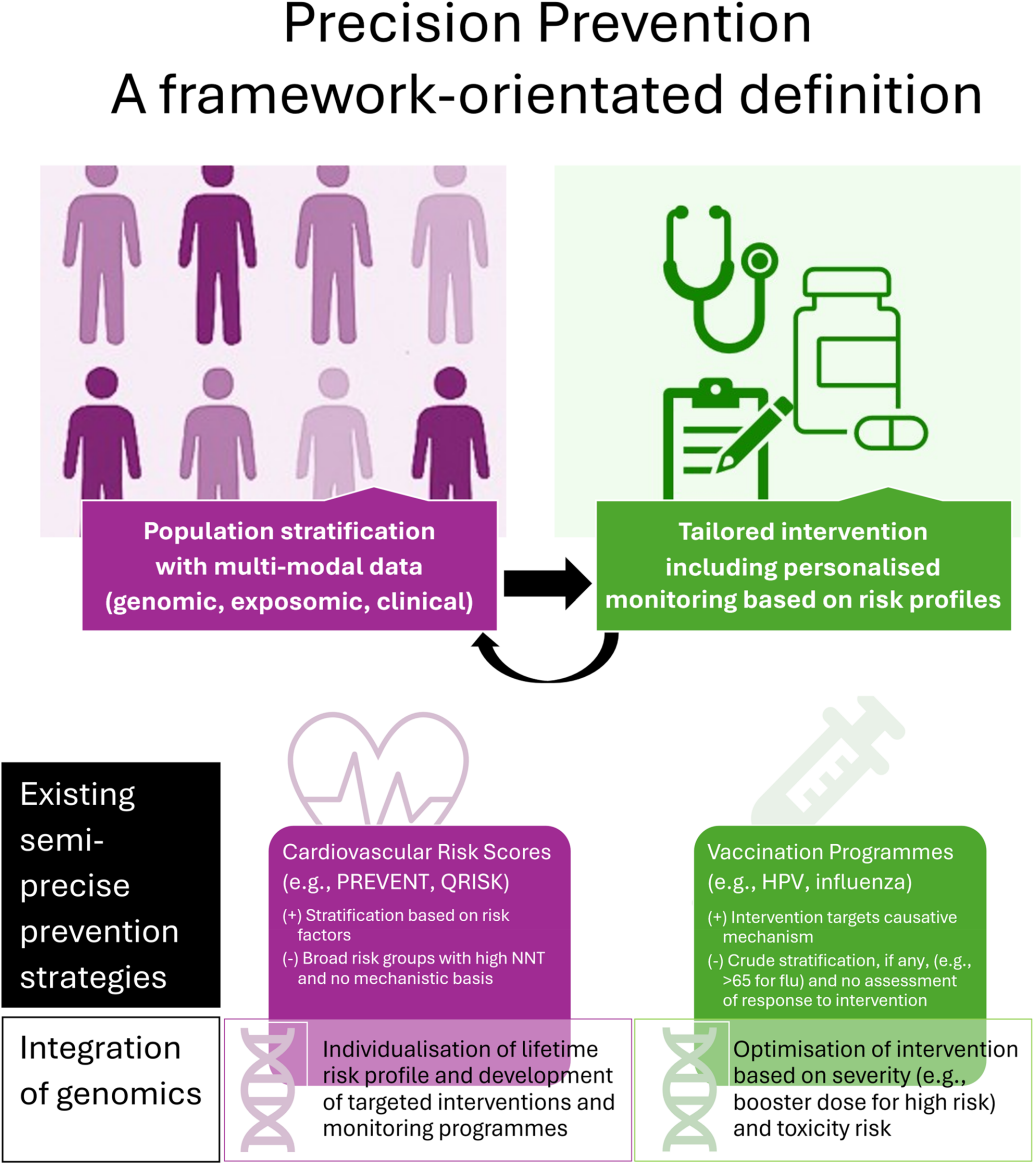
Conceptual framework for precision prevention with reflection in existing semi-precise strategies. The model combines precision population stratification (purple) and tailored interventions (green) in a cyclical process, in which each step informs the next and progressively improves precision. Cardiovascular risk scores illustrate population stratification (purple) and integrating genomics would refine lifetime risk prediction and guide novel, individualised therapies. Immunisation programmes exemplify precision intervention (targeting the cause of disease before onset) but currently lack methods to identify of high-risk subgroups who could benefit from optimised interventions

Precision prevention represents another critical evolution in healthcare, which involves a fundamental reorientation in the logic of intervention from treating a disease after symptom onset to intercepting the pre-disease processes at the level of its causal pathways. Such an approach truly individualises aspects of the healthcare cycle: risk stratification, trajectory based dynamic monitoring, and risk-proportionate intervention. Mechanism-based stratification of risk that integrates genomic, molecular, behavioural, socioeconomic and environmental data, should be sufficiently robust to distinguish individuals with similar phenotypes but different underlying biological drivers of disease. Once stratified, precision prevention should deliver proportionate interventions calibrated to an individual’s mechanistic risk profile and evolving disease trajectory complementing existing generalised public health pathways (Fig. 1).

**Fig. 2:**
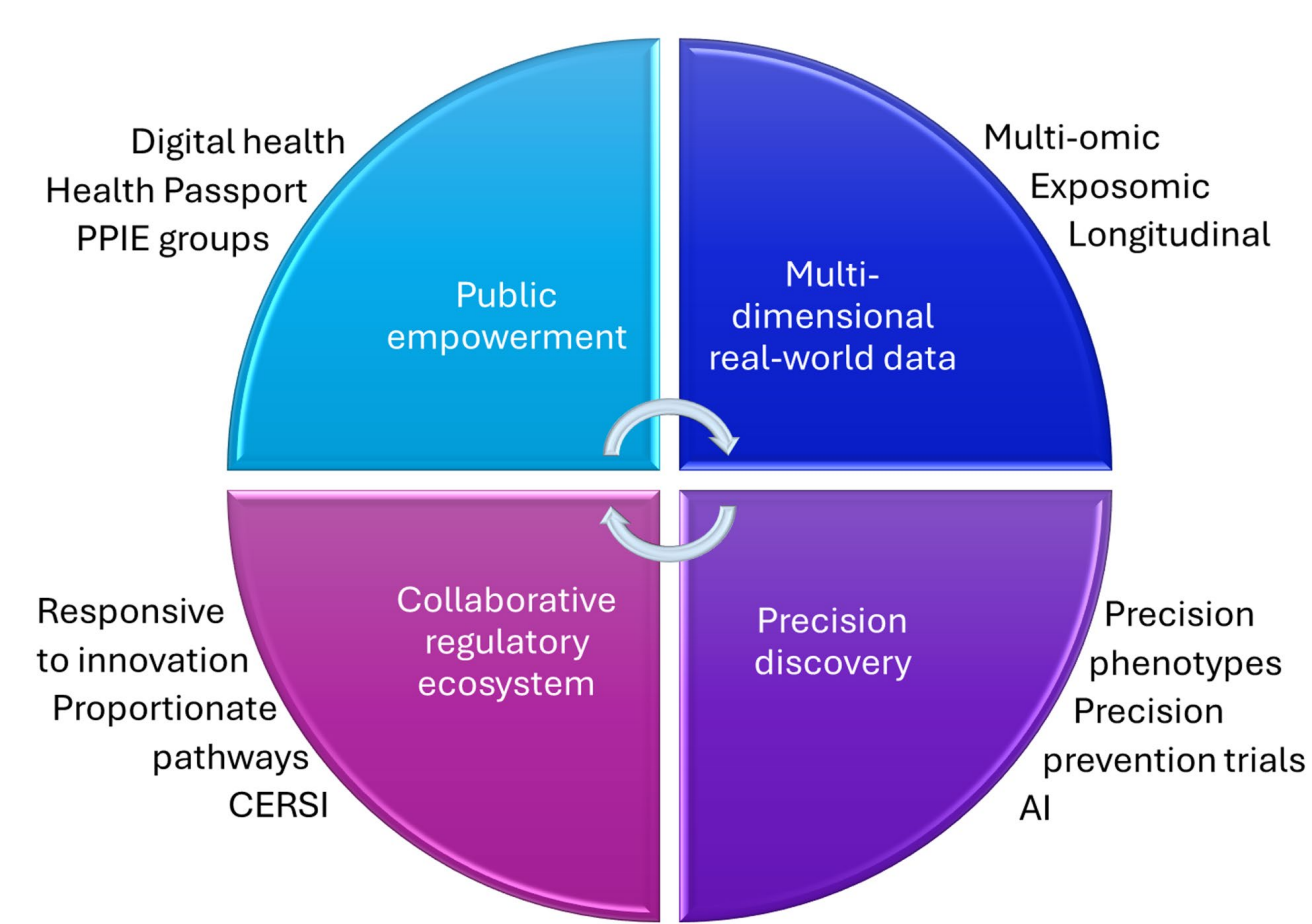
The pillars for the delivery of precision prevention within existing health systems. AI: Artificial Intelligence, CERSI: Centres of Excellence in Regulatory Science and Innovation, PPIE: Public and Patient Involvement and Engagement

The imperative for precision prevention is underscored by the limitations of current public health strategies, which predominantly target non-specific risk factors (e.g., blood pressure, lipids at the population level). The limitations of such broad approaches are evident in the increasing prevalence of the defining health challenges of our era: cardiovascular disease and cancer [1, 2]. Not surprisingly, there is significant global interest by policy makers to embrace precision prevention to alleviate the burden on healthcare systems of an aging multimorbid population [3–5]. The UK has recently unveiled a 10-year Health Plan, proposing three fundamental shifts in the National Health Service (NHS) emphasising community care, technology (genomics and artificial intelligence, AI) and prevention [6]. While genomics -in this work genomics refers to a broader term including downstream multi-omic layers and gene-environment interactions- and AI hold exceptional promise in elucidating the pathophysiology of disease, their clinical translation remains limited. The imbalance between the abundance of genomic data and the scarcity of actionable, mechanistically grounded biomarkers underscores the need to refine methodological frameworks and implementation strategies to fully harness genomics for targeted, evidence-based interventions across the population. Funding streams are required to move away from disproportionately favouring treatment-focused research with established endpoints, thereby potentially hampering the development of precision prevention [7]. AI, real-world data, precision prevention trials and regulatory science will be key enablers in the process (Fig. 2).

Precision prevention promises to transform society by shifting healthcare from reactive treatment to proactive maintenance of wellness, helping the NHS transition from managing sickness to practicing prevention. Over a longer term, this will drive a reallocation of economic value from late-stage treatment to early prediction and intervention. In this context, the workshop held on 5th March 2025 by the UK Pharmacogenetics and Stratified Medicine Network (UKPSMN) and the British Pharmacological Society (BPS) to explore the evolving field of precision prevention was especially timely and relevant. Stakeholders from academia, industry, NHS and regulatory bodies elaborated on key issues and novel approaches for the delivery of precision prevention. The opinions within this paper have been inspired by that workshop, synthesising perspectives shared during the talks and break-out sessions into a coherent set of suggestions for transforming precision prevention into actionable health strategies.

## Main text

### Artificial intelligence and precision prevention

AI can model health data at a scale and complexity which may exceed the capabilities of traditional statistical approaches, positioning them as indispensable tools for advancing precision prediction and prevention. These AI systems supported by increasing computational power can now integrate and analyse large, heterogenous datasets that encompass both stable (DNA) and dynamic (molecular and clinical) markers mostly associated with non-linear relationships. In parallel, digital technologies are now widely used to capture data on environmental exposures, social determinants of health and behavioural patterns. Continuous, real-time monitoring through wearables and smartphone applications have the potential to provide a granularity and temporal resolution of the exposome that conventional epidemiological studies have not achieved. Together, these advances allow the potential for algorithms to utilise the breadth of genomic and exposomic data with a view to generating more accurate and individualized predictions of health trajectories.

As with any tool, the potential limitations and risks of an AI approach should be recognised. Algorithm bias may arise from a number of causes, but a relevant one here is a failure of datasets to be appropriately diverse and inclusive. Algorithms trained on such datasets have a high likelihood of under-performing in individuals who were under-represented or mis-represented in that training data. Similarly, since a model is highly sensitive to its input data, a change in that data that is not biologically significant (for example a change in how a particular data type is collected or processed) can cause unexpected drop in the performance of a model.

The scale, detail and sensitivity of the data used for such models means that data privacy needs to be robustly assessed and protected [8]. As increasingly granular molecular, imaging, behavioural, and digital‑health data are linked to individuals, the risk of re‑identification and unintended disclosure grows. This raises pressing concerns about consent, data ownership, and the potential for misuse by commercial actors, including profiling, discrimination in insurance, and targeted manipulation through personalised health or marketing messages [9].

Regulatory requirements around such algorithms are primarily addressed through medical device regulation, but this is based on a legal framework which is more than two decades old, and which is still racing to catch up with how to appropriately assess risk in AI devices [10]. A frequent concern is explainability, and the extent to which the recommendations of such algorithms may be ‘black box’. This is not unique to AI-based technologies, and we successfully use many health technologies without necessarily having complete ‘explainability’ of how a drug or device works. We would rather argue that explainability is a desirable characteristic that should be provided wherever possible, but that the preeminent need is for transparency regarding the core elements of the algorithm (e.g. criteria of training set, reproducibility) and all evidence relating to its performance, including risks and likely limitations [11, 12]. Key elements for transparent reporting include but are not limited to: the biological and technical (e.g. type of AI architecture employed) rationale for its function; the training data used (including origin, and detailed description of the population diversity); all test datasets used (including detailed descriptions of those datasets and highlighting any test populations in which it performed less well than expected); any conditions required for safe operation, including technical infrastructure or training requirements; any requirements for monitoring of performance drift and model updating [13]. In summary, developers of decision-support tools must be able to clearly articulate the logic, assumptions, and data underlying their models, while end-users should possess sufficient understanding to critically assess the results and comprehensively communicate those to patients and the public. Emerging frameworks, such as PROBAST + AI, can support the critical appraisal of the quality, risk of bias and applicability of prediction models [14], while tools such as TRIPOD + AI can be used to assess transparent reporting [15]. Only with such safeguards can we take advantage of AI’s full potential as a trustworthy and equitable enabler of precision prevention.

### Precision prevention in a data-driven world

#### Causal genomic biomarkers in disease definition

The genome represents a well characterised molecular component, supported by a vast body of data and recognised for its central role in causal pathways. The power of genomics in defining precise disease phenotypes is exemplified in monogenic and high-penetrance conditions (e.g., *BRCA* mutations in cancer, *SMN* mutations in spinal muscular atrophy), where the genotype-phenotype relationship is robust and readily interrogated. In contrast, this paradigm becomes less effective in multifactorial diseases, where penetrance is lower, heritability is often modest, and heterogeneity produces non-random clusters of co-occurring conditions, all of which limit the predictive utility of genome data analysed with contemporary methods. Genomic tools such as polygenic risk scores (PRS) have demonstrated potential for clinical use in predicting certain diseases (e.g., coronary heart disease, type 2 diabetes and breast and prostate cancers), but they have yet to reach the expected level of risk discrimination and classification [16, 17]. Linking genomic variation directly to higher-order clinical symptoms necessitates consideration of the molecular layers of the transcriptome, proteome, metabolome and other -omic strata. However, the influence by secondary cascades, feedback loops, and compensatory mechanisms can obscure causal inference without the appropriate tools. Moreover, the complexity of genome–exposome interactions introduces a further layer of individual specificity. While the exposome plays a decisive role in shaping disease trajectories and refining precision risk prediction, its inherently dynamic and context-dependent nature impedes the generation of robust, generalisable evidence. Although genetic information places hard constraints on which mechanistic pathways are even possible, standard analytic tools and methodologies measure statistical associations with only a small (if any) subset of these dependencies reflecting genuine causality. Mendelian randomisation shows promise in strengthening causal interpretation by leveraging genetic variants as instrumental variables, but outputs remain sensitive to the quality of input data, assumptions about how genetic variants relate to the exposure and outcome and inability to fully account for multiple mechanistic aetiologies, underscoring the need for careful design, transparent reporting and cautious interpretation of results [18].

The main advantage of genomic risk assessment in precision prevention lies in its stability across a population and a person’s lifetime, offering a consistent indicator of susceptibility, with the ability to predict future healthcare needs (e.g. tailored monitoring before disease onset) based on allele frequencies in a randomly mixing population. Genomics-based markers are therefore best suited for primary risk prediction and population stratification. However, this stability also introduces challenges: cryptic (latent or pre-clinical) cases [19] may contaminate control groups, reducing predictive accuracy. While there is no easy way to eliminate the impact of cryptic cases, several strategies can reduce their effect. Linking participant data to registries and electronic health records (EHRs) enables longitudinal reclassification as new diagnoses emerge [20]. Replacing binary case/control labels with probabilistic disease likelihoods allows uncertainty to be modelled explicitly [21]. Rigorous control vetting (excluding individuals with early signals or borderline biomarker profiles) can further minimise contamination. Above all, carefully designed inclusion criteria, combined with adaptive frameworks that identify the crucial role of latent risk in disease progression, including age of disease onset as discussed below, will help maintain precision and interpretability in downstream analyses.

A clinical example of genome-based risk calculation in precision prevention is pharmacogenomics, which in pre-emptive testing can predict a person’s response to a drug due to variation in the genes involved in the pharmacokinetics and pharmacodynamics of the drug, preventing the occurrence of iatrogenic diseases. For example, *CYP2C19* poor metabolisers are at increased risk of adverse drug reactions and inefficacy depending on the medicine: heart failure with mavacamten and secondary stroke clopidogrel, respectively [22, 23]. Most actionable information derived from pharmacogenomics refers to one gene (and occasionally multiple variants within that gene), at present, identifying a small, but precise, cohort of the population. This approach has been shown to prevent detrimental effects driven by exposure to certain drugs in genetically defined sub-sets of the population [24], but there is room for improvement. In the future, with the increasing use of whole genome sequencing, it will be possible to assess the variation across the whole genome, and incorporate this into risk prediction models. Clearly, environmental and clinical factors can also play a role in predisposition to poor response (efficacy or safety), and these will need to be assessed in multimodal algorithms together with the genetic factors.

#### The underrated role of precision phenotypes

Many studies link rich molecular datasets to crude clinical labels or broad disease outcomes (often categorised by symptomatic presentation rather than mechanisms/pathways of pathogenesis). For example, most genomic investigations, including candidate gene studies and genome-wide association studies (GWAS) rely on binary case–control designs where outcomes are determined by ICD-10 classifications. While this approach can intuitively connect molecular discoveries to clinically recognisable endpoints as defined in EHRs, it treats these diagnostic categories as immutable and explicit. Such an approach fails to recognise that at the systems level, causal pathways are shared across multiple tissues and organ systems, meaning that disruption of a single pathway may manifest as multiple, apparently distinct diseases and a single disease may arise through multiple mechanisms. As a result, such studies may ultimately compound existing limits in knowledge rather than expand them.

No matter how sophisticated the molecular measurements are, if the corresponding phenotypes are imprecise, the generation of mechanistic insight will be constrained. Current systems of clinical classification aggregate biologically distinct processes under the same diagnostic code, masking heterogeneity in aetiology and preventing meaningful linkage between molecular signatures and causal disease mechanisms. This is evidenced by the Atlas for Health, which analysed health records between 1998 and 2020 from the total population of England (56,939,935 people) and examined patterns of disease co-occurrence. Among 100 canonical diseases, chosen to represent different organs and pathologies, computational mapping of ICD-10 diagnosis codes identified 4,166 non-randomly associated disease pairs occurring in at least 5% of individuals. Notably, one in four of these disease pairs were reported as novel by expert clinicians, highlighting how current diagnostic categories may obscure biologically meaningful structure and offering a framework to explore the molecular basis of previously unrecognised phenotypic combinations (Hemingway, unpublished data presented at the workshop).

Addressing this limitation requires a fundamental shift towards precision phenotyping -the systematic redefinition of disease states ultimately using biologically grounded, data-driven approaches. Instead of imposing phenotype-only diagnostic labels, we must allow molecular data to define new, mechanistically coherent subtypes. Early progress toward such molecular redefinition is already evident in psychiatry. For example, unsupervised clustering has been used to identify molecular subtypes of schizophrenia with distinct transcriptomic signatures, as well as transdiagnostic groupings across psychiatric conditions that align more closely with genetic architectures than traditional categories [25–28]. Genomic classifications advance precision phenotyping, but lack of external replication and inconsistent findings in well-powered studies discourage further work towards clinical translation. These issues often stem from study design, such as sample type (e.g., blood vs. brain tissue), and confounders like heterogeneous public datasets varying in demographics and protocols.

More precise, data-driven definitions can be achieved when combining molecular profiles with other data sources including imaging and video data (e.g., structural and functional organ-based scans), behavioural modalities (e.g., voice and speech recordings), longitudinal organ monitoring from wearables (e.g., heart rate) and health signals from environmental sensors. However, the need of integrative modelling frameworks for analysis necessitates careful curation of data, harmonisation across modalities and transparent reporting of how each data stream contributes to the final phenotype.

A crucial but often underestimated implication of precision phenotyping lies in the construction of valid comparator groups, rather than seeking larger sample sizes at the expense of validity. In high quality randomised clinical trials, randomisation isolates the effect of an intervention. In observational studies, robust inference requires that case groups be compared with controls in whom disease occurrence is absent—or at least minimised—through precise phenotypic definitions. At the same time, all other relevant characteristics must remain comparable. Precision phenotypes therefore not only enhance mechanistic discovery but also strengthen study validity by reducing bias.

#### Time is of the essence

Well-designed prospective studies and, in particular, randomised clinical trials, remain the gold standard for evaluating an intervention. However, challenges arise when the clinical endpoint of interest occurs only after a long latency period. This is particularly problematic in cancer prevention research, where the interval between a preventive intervention and cancer occurrence may span decades. Such timelines substantially increase cost and reduce feasibility. For this reason, precision prevention studies in oncology often focus on high-risk populations who are more likely to develop cancer within a shorter timeframe [29]. These studies also recognise that cancer occurrence represents a late stage of a much longer disease trajectory and therefore prioritise the collection of diverse biological materials to identify surrogate markers of pre-cancer states.

Central to precision prevention is viewing disease as a continuum embedded in a complex system, that offers opportunities for intervention long before clinical disease manifests. When viewed through a true systems-level understanding, disease can no longer be conceptualised solely as a discrete clinical event, but rather as a process that begins long before symptoms arise. Progression of a disease from causal mechanisms, through pre-disease states, and ultimately to symptomatic presentation represents a continuum shaped by an individual’s genetic architecture, cumulative exposures, and their complex interactions. Hence, more responsive and longitudinal measures (for example, biochemical markers, or digital tracking) are better deployed for dynamic surveillance and trajectory monitoring.

It is essential that such measures are not integrated into risk prediction frameworks as static inputs, but rather as components of adaptive strategies. In this approach, the data itself informs when/how frequently (through adaptive sampling), and what additional information should be collected (adaptive predictor selection), according to an individual’s evolving risk profile and associated uncertainty—balancing predictive accuracy with participant burden. For example, an initial model may rely on demographic (e.g., sex, height) and genetic variables for baseline stratification; where uncertainty remains high, specific biochemical, higher omic and exposomic data could then be requested via digital tools initiated to enhance predictive precision.

Real-world data play a critical role in improving the reliability, scalability, and patient-centred nature of such models. Yet, researchers must take care to align data collection with appropriate temporal considerations. Biobank samples and linked EHR data should be contextualised with respect to participant age and the epidemiological peak of the disease to ensure comparator groups are validly defined. For instance, studies investigating the genomic architecture of cardiovascular diseases may require an age threshold in the comparator group, given that clinical onset typically occurs later in life while the underlying genomic factors remain constant throughout the lifespan. Similarly having a genetic mutation may confer an increased risk of cancer at a specific age which should define participants to achieve maximum benefit from the intervention.

#### Making use of noise in complex biological systems

Traditional diagnostics operate by establishing thresholds derived from population-level distributions (for example, upper and lower limits of common blood tests) to capture variability across many individuals. This approach has the advantage of simplicity: it defines clear clinical cut-offs, is easily interpretable, and has served medicine well for decades. Precision prevention seeks to further account for this variability by attempting to separate true stochastic signals from emergent properties of complex systems. What appears as noise at the population level (be it genetic variants, epigenetic drift, microbiome dynamics, cumulative micro-exposures, or any other stochastic process) may in fact represent meaningful biology either shaping or indicating an individual’s unique risk trajectory. The challenge is therefore not only the absence of a signal(s), but the current limitations of our measurement and modelling frameworks in capturing, interpreting, and integrating these subtle but consequential processes. Whether AI can help us achieve this has not been adequately investigated yet.

### Calls to action

Translating precision prevention into routine practice requires a systemic reconfiguration of how health systems define disease states, interpret evidence of intervention benefit, deliver care in a prevention-oriented world, and conceptualise value and objectives. The shift from treating to preventing sickness requires close collaboration between all actors in the health system: research and development, clinical practitioners, industry, regulators and policymakers (Table 1).

**Table 1:** Calls to action

Stakeholder	Challenges	Calls to action	System impact
Healthcare systems	- Reimbursement models often reward treatment episodes - Prevention benefits often occur outside short financial cycles	- Develop reimbursement frameworks with delayed disease onset as a measurable outcome - Integrate precision prevention into service models and workforce planning	Reallocation of economic value from late-stage treatment to early prediction and intervention. Sustainable system capacity and improved population health.
Research community and funders	- Biomarker discovery perceived as high risk, resource-intensive - Funding streams skewed toward treatment studies with established endpoints	- Incentivise mechanistic phenotyping research - Establish long-term funding programmes spanning biomarker discovery, validation, and diagnostic development - Create shared royalty models between government, academia, and industry	Drive development of molecularly defined disease, moving beyond broad phenotype-based classifications. Mechanism focussed decomposition of broad disease labels into biologically grounded subtypes (endotypes) enables targeted preventive strategies and more efficient downstream research.
Industry	- Traditional business models centred on treatment endpoints, and favour the investigation of a new medicinal product - Prevention requires long expensive trials - Perception of regulatory and commercial uncertainty.	- Share archived trial biospecimens for multi-omics research to allow investigation of prevention associated additional label to an existing product - Invest in the development of surrogate endpoints - Build academic–industry–healthcare partnerships and support cross-sector training of next generation of translationally focussed health leaders.	Industry evolves from downstream implementer to co-creator of prevention strategies. Growth in diagnostics, biotech, and digital health sectors.
Regulators and health technology assessment bodies	- Regulatory frameworks not optimised for iterative evidence generation (e.g., multivariable biomarker panels, surrogate endpoints, longitudinal data models) - Evaluation frameworks undervalue long-term prevention due to short time horizons, discounting, and reliance on near-term endpoints	- Accept validated biomarkers as surrogate endpoints - Develop shared infrastructures linking academia, industry, and government to co-create standards - Enable adaptive, data-driven regulatory models - Commercially incentivise label expansion from treatment to prevention - Adapt value assessment models to capture delayed disease onset, reduced health-system utilisation, and quality-of-life gains - Reform implementation of QALY-based approaches to better reflect long-term benefit (e.g., reduced social care costs, reduced welfare burden, increased economic productivity)	Alignment of regulatory and reimbursement systems with prevention-focused innovation, enabling earlier intervention, sustainable healthcare financing, and recognition of long-term societal and economic value.

A fundamental pillar of precision prevention relies on utilising longitudinal and multivariable biomarker models to infer chronic disease risk and redefine disease states through molecular definitions. Biomarker-focused research is often deprioritised by funders due to perceived cost, complexity, and uncertain short-term returns; consequently, precision prevention requires strategic, longer-term funding initiatives. Spanning discovery, validation, and diagnostic creation, these initiatives need mechanisms for shared royalties between government, academic, and private stakeholders to bridge the existing gap.

At the level of care delivery, precision prevention increases complexity, requiring the integration of genomic, biomarker, and socio-environmental data into routine practice within an already constrained system. This demands not only robust digital infrastructure but also a workforce capable of interpreting and acting upon increasingly complex risk information.

Finally, at the level of conceptualisation of value (and unlike therapeutic interventions typically evaluated against discrete, validated clinical endpoints), the challenge is that prevention operates across longer time horizons and relies on probabilistic risk modification, and this longer time-frame that delays disease onset, reduces healthcare utilisation, improves quality of life and improves economic productivity needs to be taken into account in economic modelling.

## Conclusions

Precision prevention has the potential to transform healthcare from a population-level, risk-factor–oriented practice to a mechanistically grounded, individually adaptive approach. By identifying and modulating risk at the causal pathway level, it could enable longer, healthier lives and reduce the burden of chronic disease. Careful integration of AI systems in healthcare, transformation of discovery methodologies to reflect precision inputs and deliver precision outputs, maintaining a holistic use of all available knowledge, and aligning all stakeholders toward a common goal are among the many steps needed to reshape the public health prevention space. Realising this promise, however, will require a fundamental shift in mindset among stakeholders to adapt traditional approaches to the evolving capabilities of science and technology.

## Data Availability

No datasets were generated or analysed during the current study.

## Abbreviations

NHS: National Health Service
UKPGx: UK Pharmacogenomics
BPS: British Pharmacological Society
AI: artificial intelligence
PRS: polygenic risk score
EHR: electronic health records
PGx: pharmacogenomics
GWAS: genome-wide association study
